# Studies of Intra-Chain and Inter-Chain Charge Carrier Conduction in Acid Doped Poly(3,4-ethylenedioxythiophene) Polystyrene Sulfonate Thin Films

**DOI:** 10.3390/ma18194569

**Published:** 2025-10-01

**Authors:** Ayman A. A. Ismail, Henryk Bednarski, Andrzej Marcinkowski

**Affiliations:** 1 Centre of Polymer and Carbon Materials, Polish Academy of Sciences, M. Curie-Skłodowskiej 34, 41-819 Zabrze, Poland; ayman.abdallahahmedismail@polsl.pl (A.A.A.I.);; 2 Joint Doctoral School, Silesian University of Technology, Akademicka 2a, 44-100 Gliwice, Poland

**Keywords:** PEDOT:PSS, optical conductivity, electrical conductivity, percolation, effective medium approximation

## Abstract

Poly(3,4-ethylenedioxythiophene): polystyrene sulfonate (PEDOT:PSS) is a conductive water-processable polymer with many important applications in organic electronics. The electrical conductivity of PEDOT:PSS layers is very diverse and can be changed by changing the processing and post-deposition conditions, e.g., by using different solvent additives, doping or modifying the physical conditions of the layer deposition. Despite many years of intensive research on the relationship between the microstructure and properties of these layers, there are still gaps in our knowledge, especially with respect to the detailed understanding of the charge carrier transport mechanism in organic semiconductor thin films. In this work, we investigate the effect of acid doping of PEDOT:PSS thin films on the intra-chain and inter-chain conductivity by developing a model that treats PEDOT:PSS as a nanocomposite material. This model is based on the effective medium theory and uses the percolation theory equation for the electrical conductivity of a mixture of two materials. Here its implementation assumes that the role of the highly conductive material is attributed to the intra-chain conductivity of PEDOT and its quantitative contribution is determined based on the optical Drude–Lorentz model. While the weaker inter-chain conductivity is assumed to originate from the weakly conductive material and is determined based on electrical measurements using the van der Pauw method and coherent nanostructure-dependent analysis. Our studies show that doping with methanesulfonic acid significantly affects both types of conductivity. The intra-chain conductivity of PEDOT increases from 260 to almost 400 Scm^−1^. Meanwhile, the inter-chain conductivity increases by almost three orders of magnitude, reaching a critical state, i.e., exceeding the percolation threshold. The observed changes in electrical conductivity due to acid doping are attributed to the flattening of the PEDOT/PSS gel nanoparticles. In the model developed here, this flattening is accounted for by the inclusion shape factor.

## 1. Introduction

Conducting polymers have attracted extensive attention over the past few decades due to their unique combination of electrical conductivity and mechanical flexibility, positioning them as promising materials for a wide range of applications, including organic electronics, energy storage, sensors, and flexible optoelectronics [1,2,3]. Among these, poly(3,4-ethylenedioxythiophene): polystyrene sulfonate (PEDOT:PSS) stands out as one of the most widely studied and commercially used conductive polymers due to its excellent processability, high transparency in the visible range, environmental stability, and tunable electrical properties [4,5,6]. PEDOT:PSS thin films have become crucial components in devices such as organic photovoltaics, light-emitting diodes, touch screens, and thermoelectric devices [7,8,9,10]. Typically, PEDOT:PSS consists of positively charged PEDOT chains stabilized by negatively charged long PSS chains, where the intrinsic conductivity is often limited by the insulating nature of PSS and the degree of phase segregation between the two components [10,11,12]. PEDOT:PSS aqueous dispersions are commercially available in a wide range of electrical conductivities, which are achieved by varying the PEDOT to PSS ratio. The electrical conductivity of PEDOT:PSS films is known to be highly sensitive to their microstructure, morphology, and doping level [13,14,15]. Enhancing it is a major research focus, often achieved by direct solvent modification as well as films post-treatment with solvents, acids, or other dopants that modify the charge density, film morphology, and, in consequence, the charge transport pathways [16,17,18,19]. In particular, acid doping has been shown to significantly improve conductivity by increasing the degree of order in the PEDOT reach domains and enabling the removal of excess PSS, which results in enhanced carrier mobility and percolation pathways [20,21,22,23]. Despite numerous studies demonstrating conductivity improvement upon the addition of specific solvents or acid doping, the mechanisms behind this effect in PEDOT:PSS films remain a matter of debate [24,25,26,27].

Charge conduction in conducting or semiconducting polymers can be broadly categorized into two contributions: intra-chain and inter-chain transport. Intra-chain conduction refers to charge transport along the conjugated polymer backbone, where carriers move through delocalized π-electron systems. In contrast, inter-chain conduction involves charge hopping or tunneling between adjacent polymer chains, aggregates or grains, a process influenced by the degree of chain packing, crystallinity, and morphology of the film [28,29,30,31,32,33]. The relative contributions of these two transport mechanisms are critical for optimizing device performance but are often conflated in experimental measurements. Several models have been proposed to describe electrical conduction in films of these conducting polymeric materials, including hopping conduction, percolation theory, and effective medium approximation (EMA) [32,33,34,35,36,37]. The generalized effective medium theory (GEMT) has proven to be a valuable framework for describing conductivity in composite materials, including polymer blends and doped conducting polymers, as it accounts for the conductive contributions from multiple phases within the material [37,38,39,40,41]. In the context of PEDOT:PSS, Stöcker et al. [39] used the GEMT method to separate the contributions from percolation between sites of highly conductive PEDOT:PSS complexes from the less conductive PSS matrix. In a later approach, Bednarski et al. investigated the effect of the PEDOT to PSS ratio on the optical properties of PEDOT:PSS thin solid films and developed a composition-dependent optical model of PEDOT: PSS based on the chemical mixture approximation [40]. Furthermore, they related the optical conductivity of free carriers derived from PEDOT to the electrical conductivity of PEDOT:PSS within the GEMT framework [40]. In particular, it was emphasized that optical techniques are sensitive to local conductivity in PEDOT-rich regions. However, electrical measurements allow for the determination of global conductivity, which contributes much more to the resistive PSS. This latter approach also lays the foundation for a non-contact method for determining the electrical conductivity of PEDOT:PSS films.

The aim of this work is to deepen the knowledge on intra-chain and inter-chain conductivity of charge carriers in acid-doped PEDOT:PSS thin films by developing a model of their optical and electrical properties that treats PEDOT:PSS as a nanocomposite material. Since most studies on improving the conductivity of PEDOT:PSS films using acids rely on post-deposition treatments with strong acids, here we focus on the less-explored method of adding a diluted acid directly to the aqueous PEDOT:PSS dispersion before deposition of the film on the substrate. For this purpose, we will use methanesulfonic acid (MSA), as Song et al. [28] found that this acid, at high concentrations, is ideal for post-deposition treatments to increase the conductivity of films without damaging plastic substrates such as polyethylene terephthalate. Furthermore, our approach quantitatively attributes the high conductivity of free carriers originating from PEDOT-rich cores to intra-chain carrier transport, using parameters derived from the optical Drude-Lorentz model, while the weaker inter-chain conductivity, between PEDOT-rich grains, is characterized by electrical conductivity determined using the van der Pauw technique and morphology-dependent coherent analysis. In addition, we critically discuss all assumptions made, presenting solid physical justification for them.

## 2. Materials and Methods

### 2.1. Materials

Poly(3,4-ethylenedioxythiophene): polystyrene sulfonate (PEDOT:PSS), a conductive polymer dispersion (Clevios™ HTL Solar, Heraeus), was purchased from Ossila (Product Code: M124). Methanesulfonic acid (98%, Fluka brand) was used as the doping agent. Isopropanol (IPA) was used for substrate cleaning. Standard microscope glass slides served as the substrate for thin film deposition.

### 2.2. Preparation of PEDOT: PSS-MSA Thin Films and Characterization Methods

To investigate the influence of methanesulfonic acid (MSA) doping on the properties of PEDOT:PSS thin films, a series of doped formulations were prepared using a 1 M MSA solution, derived from 98% concentrated MSA. A fixed volume of PEDOT:PSS dispersion (165 µL) was mixed with incremental volumes of the MSA solution: 0, 1, 2, 3, 4, 5, 6 and 7 (±0.1) µL. This resulted in final MSA concentrations in the PEDOT: PSS dispersion of 0.0 M, 0.006 M, 0.012 M, 0.018 M, 0.024 M, 0.030 M, 0.036 M, and 0.042 M, respectively. Each time solution was continuously stirred to ensure complete mixing, followed by homogenization using a roll mixer. Glass microscope slides were used as substrates and were thoroughly cleaned with isopropanol and air-dried at room temperature to ensure surface cleanliness and enhance film adhesion. For film deposition, 20 µL of each time PEDOT:PSS-MSA solution was deposited onto the center of a prepared substrate. A two-step spin-coating process was employed: first at 500 rpm for 3 s evenly distribute the dispersion, followed by a second spin step at 3000 rpm for 3 s to produce smooth, continuous films. The coated films were then baked at 120 °C for 5 min to remove residual solvents. Electrical contacts were formed by applying silver paste to the film surfaces. Finally, a post-annealing step was conducted at 65 °C for 5 min to ensure stable adhesion and enhance the conductivity of the contacts. A summary of the film preparation parameters and MSA concentrations is presented in Table S1.

For sample characterization, the following methods were used:

AFM imaging was performed with a Dimension ICON system (BRUKER, Billerica, USA) in soft tapping mode under ambient conditions, using doped silicon cantilevers (PPP-NCH-10, NANOSENSORS) with a stiffness of 42 N/m. Surface morphology was analyzed using Nano Scope Analysis 1.9 software.

Optical measurements were recorded using a JASCO V-570 UV–Vis–NIR spectrometer (JASCO Corporation, Tokyo, Japan).

Electrical conductivity and Hall effect measurements were carried out using the HMS-TT Fast Hall Station (Lake Shore Cryotronics Inc., Westerville, OH, USA) under a reversible magnetic field of 1 T to study electrical parameters.

### 2.3. Measurements and Uncertainties

The direct measurements conducted for this work were performed using high-end, high-resolution measurement and research equipment and proprietary computer software. For example, the claimed resolution of the atomic force microscope is in the angstrom range. Electrical measurements were performed with a signal-to-noise ratio greater than 100 and a sampling rate of 100. The claimed spectral resolution and sensitivity of the JASCO spectrophotometer are also very high. The key parameter of layers is their thickness, because the accuracy of its determination influences the accuracy of other physical quantities that depend on it. In this work, the layer thickness was determined based on AFM studies. The determined thicknesses of all tested layers ranged from 100 to 200 nm, and their uncertainty was statistically estimated at +/− 15% (average of 39 images taken from 21 different films, each containing mostly three measurement points). To calculate the standard deviations of the multivariate dependent variables, error propagation analysis was performed, neglecting correlations between variables. The goodness of fit was assessed using the mean square error (MSE).

## 3. Results and Discussion

### 3.1. Theoretical Background

#### 3.1.1. Dielectric Model of PEDOT:PSS Thin Films—Nanocomposite Approach

A model of the dielectric function of composite materials such as PEDOT:PSS [40] or P3HT [38], combining the properties of both components and dependent on the composition, was developed by Bednarski et al. For this purpose, in [38,40], the chemical mixture approximation (CMA) was used. In this approach, the complex dielectric function of the composite material, $\varepsilon_{PEDOT:PSS}$, is expressed by a sum of weighted contributions, as follows [38,40]:

(1) $$\varepsilon_{PEDOT:PSS}=\left(1-f\right)\varepsilon_{PSS}+f\varepsilon_{PEDOT}$$

where $\varepsilon_{PSS}$ is the dielectric function of a majority material, $\varepsilon_{PEDOT}$ is the dielectric function of a minority material (inclusion) and f is its volume fraction. In [40], it was argued that in the case of PEDOT: PSS water dispersions, the water-soluble and hydrophilic PSS is always the majority material, ensuring their temporal stability. Moreover, PSS acts as a doping agent, and both the short PEDOT oligomers and the PSS long chains interact strongly electrostatically, forming the PEDOT:PSS complex. This fact supports the application of the CMA to the PEDOT:PSS system. So far, we have not considered explicitly the anisotropy of the material, but it is known that PEDOT:PSS layers exhibit uniaxial optical anisotropy, see, e.g., [40] and references therein. Fortunately, with the optical axis perpendicular to the layer surface, this is not a difficult task. In this case, the dielectric function tensor is still diagonal, with an in-plane ordinary component $\varepsilon_{xx}=\varepsilon_{yy}=$ $\varepsilon_{∥}$ and an out-of-plane extraordinary component $\varepsilon_{zz}=\varepsilon_{⊥}$. Therefore, to characterize the dielectric properties of the PEDOT:PSS layer, a two-component dielectric function is needed. Analogically, for the electrical conductivity, the in-plane and out-of-plane electrical conductivity should be distinguished.

In this paper, we abandon CMA and try to find a more physically justified approximation describing the dielectric properties of PEDOT:PSS thin films considered as a nanocomposite material. It turns out that the linear EMA (similar to Equation (1)) dependence was derived for very thin multilayer structures parallel to the layer surface, i.e., described by the ordinary component of the dielectric function $\varepsilon_{∥}$, see [41] and references therein, while the corresponding extraordinary component can be written as follows:

(2) $$\varepsilon_{⊥}={\left(\frac{1-f}{\varepsilon_{PSS}}+\frac{f}{\varepsilon_{PEDOT}}\right)}^{-1}$$

To complement this, and to better adapt to the nanostructure and morphology of PEDOT:PSS layers, we note that these relations also constitute a limiting case of the general Bruggerman mixing approximation for two-phase composite materials with ellipsoidal inclusions [42,43], here written for PEDOT:PSS treated as a nanocomposite material:

(3) $$\frac{\left(1-f\right)\left(\varepsilon_{PSS}-\varepsilon_{PEDOT:PSS}\right)}{L\varepsilon_{PSS}+\left(1-L\right)\varepsilon_{PEDOT:PSS}}+\frac{f\left(\varepsilon_{PEDOT}-\varepsilon_{PEDOT:PSS}\right)}{L\varepsilon_{PEDOT}+\left(1-L\right)\varepsilon_{PEDOT:PSS}}=0$$

where *L* is the inclusion depolarization (shape) factor. As can be seen from the direct calculation, after substituting *L* = 0, this relation transforms into Equation (1), assuming that the sum of the volume fractions of both inclusions is 1. In turn, after substituting *L* = 1, Equation (3) can be transformed into Equation (2). It should be noted that Equation (3) is actually a system of three equations for the three components of the dielectric function, linked by the condition that the corresponding depolarization factors along the principal axes of the ellipsoid must sum to 1. In general, *L* takes values from 0 to 1. *L_x_ = L_y_ = L_z_* = 1/3 corresponds to a spherical shape of the inclusion, *L_x_ = L_y_* = 0 and *L*_z_ = 1 corresponds to an extremely oblate ellipsoid in the *xy* plane. On the other hand, an ellipsoidal inclusion in the shape of an elongated cigar along the *z* axis has *L_x_ = L_y_* = 1/2 and L_z_= 0 [44,45].

Below we present arguments in favor of adopting such a lamellar dielectric model for PEDOT:PSS layers, based on Equation (3) and the generally accepted knowledge about the morphology, microstructure and nanostructure of such layers. Namely, PEDOT:PSS gel particles in aqueous dispersion consist of a hydrophobic PEDOT-rich core and a surrounding hydrophilic PSS-rich shell with diameters of 12–19 nm, respectively, after changing the solution pH from 1.7 to 9 [46]. Therefore, thin PEDOT:PSS layers composed of such closely packed spherical gel particles will ultimately form a multilayered, lamellar microstructure. More precisely, starting from the substrate on which the film is deposited, we will have alternating layers: a PSS layer composed of PSS shells of gel particles, then a PEDOT-rich layer composed of PEDOT-rich cores. Then another PSS layer composed of two PSS shells (derived from the gel particles of the first and second layers), and so on. In this way, the dielectric lamellar model of the PEDOT:PSS nanocomposite can be described by Equation (3) for oblate spheroids whose equatorial plane is oriented parallel to the layer surface, with depolarization coefficients close to their limiting values, i.e., 0 and 1. Since, these films are relatively thin, with a thickness of a few nm, which results from the size of the PEDOT:PSS gel nanoparticles.

In Section 3.2 this equation will be used to determine the optical conductivity of acid doped PEDOT:PSS thin films. For this purpose, we will refer to the general relationship between dielectric function and electrical conductivity, which arises from Maxwell’s equations in a material medium.

The next step is to parameterize $\varepsilon_{PSS}$ and $\varepsilon_{PEDOT}$, which we will postpone until Section 3.3. At this point, we briefly mention that, in order to maintain continuity with the previous work [40], we will use exactly the same parameterization of the optical model for PEDOT:PSS. In particular, the dielectric function of PSS will be approximated by Tauc-Lorentz oscillators, while the dielectric function of PEDOT will be approximated by Drude-Lorentz oscillators.

#### 3.1.2. Model of Electrical Conductivity of PEDOT:PSS Thin Films—Composite Approach

In relating optical properties to electrical conductivity in [39,40] the GEMT equation were involved [37,38,39,40,43]. The GEMT relates electrical conductivity of a well-conducting material, $\sigma_{c}$, and a more resistive material, $\sigma_{r}$, with the conductivity of a composite media, $\sigma_{M}$, by the following equation [44]:

(4) $$\frac{\left(1-f\right)\left(\sigma_{r}^{\frac{1}{w}}-\sigma_{M}^{\frac{1}{w}}\right)}{\sigma_{r}^{\frac{1}{w}}+\frac{1-f_{c}}{f_{c}}\sigma_{M}^{\frac{1}{w}}}+\frac{f\left(\sigma_{c}^{\frac{1}{w}}-\sigma_{M}^{\frac{1}{w}}\right)}{\sigma_{c}^{\frac{1}{w}}+\frac{1-f_{c}}{f_{c}}\sigma_{M}^{\frac{1}{w}}}=0$$

where *f_c_* is the (critical) percolation volume fraction of the conducting inclusion material, and the model parameter w is the critical exponent that is usually fitted to the experimental data. This equation is valid for both AC and DC conductivity.

As can be seen, Equation (4) for w = 1 is essentially the Bruggerman equation for electrical conductivity [42,44], with its functional form corresponding to Equation (3). The only difference is that instead of the inclusion shape factor *L*, *f_c_* is used, which is the critical volume fraction of the conductive component at which the probability of forming an infinite conductive path in the composite material is 1.

It should also be noted that when applying Bruggerman homogenization to describe the optical conductivity of PEDOT:PSS layers, we use Equation (4) for *L* = 0 for the following four reasons. Namely, (i) the electric field of a light wave oscillates in a plane parallel to the layer surface when light strikes it perpendicularly. And (ii) even below the percolation limit, in-plane optical conductivity can be determined. (iii) As can be seen, for L = 0, Equation (4) corresponds to a parallel combination of the average resistances of the PEDOT- and PSS-rich components. Finally, (iv) individual PEDOT:PSS gel particles in the layer cannot be distinguished by optical studies in the wavelength range of light from 400 to 2000 nm, because these studies only allow for resolving morphological details of the microstructure of the layers with dimensions comparable to the wavelength of the light used. Therefore, in this case, practically only the contribution of the highly conductive PEDOT is visible, which we identify as intra-chain conduction.

**Figure 1:**
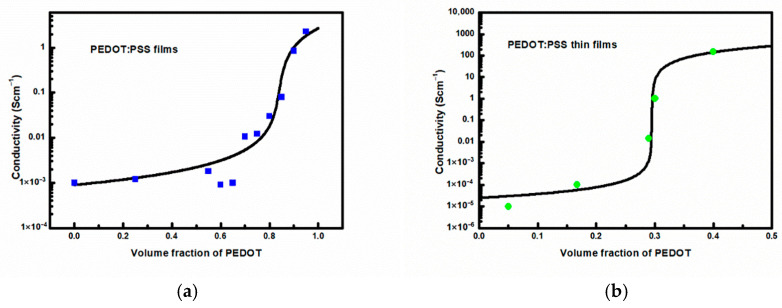
Bruggerman effective medium approximation to the electrical conductivity for data of: (**a**) Stöcker et al. [39] (squares) and (**b**) Bednarski et al. [40] (circles), as a function of PEDOT volume fractions. Calculated using Equation (4) with *w* = 1.

By reducing the GEMT equation to the Bruggerman approximation for effective conductivity, we formally reduce the flexibility, technically speaking, of Equation (4) by replacing the fitted parameter w with a constant, in this particular case equal to 1. We will therefore show that this is possible even based on previously published results, without noticeable deterioration of the fit. To verify this, we used the results of the electrical conductivity of PEDOT:PSS layers as a function of the PEDOT to PSS ratio given by Stöcker et al. [39] as well as our results from [40] for several commercially available aqueous PEDOT:PSS dispersions with different PEDOT to PSS ratios. The results of these calculations are presented in Figure 1a,b, respectively, and the values of the fitting parameters are given in the figure caption. As can be seen, the description obtained using Bruggerman EMA (BEMA) is acceptable and is not noticeably worse than the description obtained using GEMT in [39,40].

### 3.2. AFM

**Figure 2:**
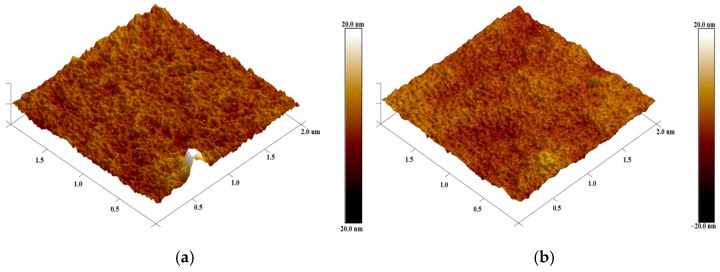
Three-dimensional surface AFM images of PEDOT:PSS films: (**a**) undoped, R_q_ = 5 nm; (**b**) doped with 0.042M of MSA, R_q_= 1.76 nm.

The surface morphology of MSA-doped PEDOT:PSS thin films was determined using atomic force microscopy (AFM). The obtained images indicate that the studied films are characterized by a slightly rough surface. This can be seen in Figure 2, which compares the surface images of the undoped PEDOT:PSS film and the most heavily doped MSA film, deposited from a solution with a molar concentration of 0.042 M.

**Figure 3:**
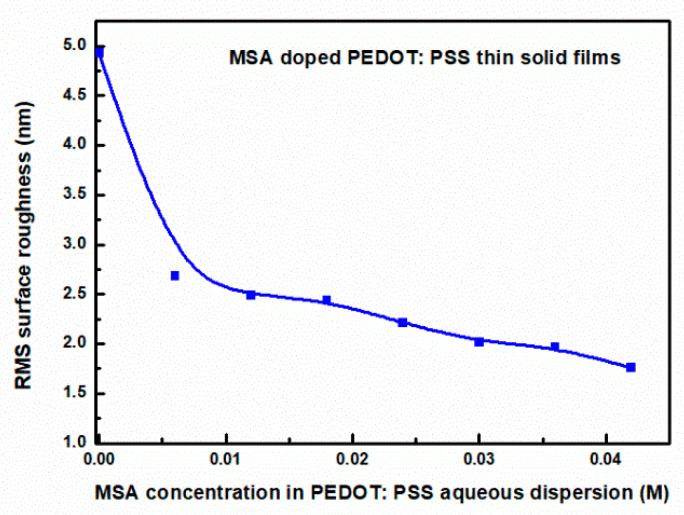
Surface roughness RMS (R_q_) as a function of MSA doping level.

A closer analysis reveals a correlation between the degree of doping and the surface roughness index Rq. The value of this index decreases with increasing doping, as shown in Figure 3. This trend is consistent with the results obtained by Jain et al. [46], who studied the particle size of a colloidal PEDOT:PSS gel in solutions with different pH values. In particular, they observed that the diameter of these gel particles decreases with increasing solution acidity. Because our MSA-doped PEDOT:PSS thin films were deposited from solutions to which MSA had been added, they formed spheres with sizes depending on the amount of acid added. Therefore, it seems natural that the surface formed by smaller-diameter spheres would have lower roughness and be characteristic of more heavily doped films, i.e., those made from solutions containing more MSA. Based on AFM studies, the thicknesses of the tested films were also determined and are presented in Table S2 in the Supplementary Materials.

### 3.3. Studies of Intra-Chain Charge Carrier Conduction in MSA Doped PEDOT:PSS Thin Films

While studying the effect of the PEDOT to PSS ratio on the optical properties of PEDOT:PSS thin films, Bednarski et al., observed a strong dependence of the optical conductivity values on the PEDOT content [40]. Furthermore, the nominal conductivity values of these films were significantly lower. These observations were explained by the core–shell structure of PEDOT:PSS micelles, from which the solid film is formed, leading them to conclude that optical techniques tend to probe local conductivity in PEDOT-rich regions. However, electrical measurements provide data on the global conductivity, which results from the much more resistive PSS [40]. Based on these findings, we go a step further and attribute this high free carrier conductivity, derived from optical conductivity, to intra-chain charge transport of PEDOT. We also note that this can also be attributed to high intra-core charge carrier transport in PEDOT-rich cores due to the presence of the extended π-electron system of stacked PEDOT chains, in which π-orbitals from adjacent chains overlap [32].

As can be concluded from the above discussion, information on the effect of MSA doping on intra-chain conductivity in PEDOT:PSS thin films can be obtained from studies of the optical conductivity of these films. From electromagnetism textbooks, it is known that the dielectric function is related by means of Maxwell’s equations to the electrical conductivity of the material being studied in the following way, e.g., see [47]:

(5) $$\varepsilon \left(\omega \right)=\varepsilon_{0}+\frac{i\sigma \left(\omega \right)}{\omega }$$

where $\varepsilon_{0}$ is the vacuum permittivity, *i* denotes the imaginary unit, and both $\varepsilon =\varepsilon_{1}+i\varepsilon_{2}$ and $\sigma =\sigma_{1}+i\sigma_{2}$ are complex numbers. Therefore, from Equation (5), the following relation between the real part of the conductivity $\sigma_{1}$ and the imaginary part of dielectric function $\varepsilon_{2}$ can be derived:

(6) $$\sigma_{1}=\varepsilon_{0}\omega \varepsilon_{2}$$

Recalling now that $\varepsilon_{2}$ is equal to the double product of the refractive index *n* and the extinction coefficient *k*, we see from Equation (6) that the term optical conductivity is fully justified. In our approach, we use the relationship given by Equation (5) to determine the optical conductivity of MSA-doped PEDOT:PSS thin films, assuming that $\varepsilon_{2}$ is approximated by Equation (1), i.e., $\varepsilon_{∥,2}=\varepsilon_{PEDOT:PSS,2}=\left(1-f\right)\varepsilon_{PSS,2}+f\varepsilon_{PEDOT,2}$, the detailed justification of which is presented above in Section 3.1.1. As shortly mentioned at the end of that section, $\varepsilon_{PSS}$ and $\varepsilon_{PEDOT}$ are parameterized by the sum of two Tauc-Lorentz oscillators, while the PEDOT dielectric function is approximated by the sum of Drude and Lorentz oscillators.

Let us now note that the linear relation expressed by Equation (6) implies a linear dependence of the conductivity $\sigma_{1}$ with respect to all components of the dielectric function $\varepsilon_{2}$. Since the Tauc-Lorentz and Drude-Lorentz dielectric function models are well known, we will not consider all them here. We will only briefly discuss the Drude oscillator to show its usefulness not only in describing the PEDOT conductivity at optical frequencies but also in determining its extrapolated value in the stationary limiting case at ω=0. Namely, the free electron dielectric function is described by the Drude oscillator as follows [25,47]:

(7) $$\varepsilon \left(\omega \right)=1-\frac{\omega_{p}^{2}}{\omega \left(\omega -i\omega_{\tau }\right)}$$

where $\omega_{p}$ is the plasma frequency and $\omega_{\tau }$ is the damping parameter. This expression can be written explicitly for its real (*Re*) and imaginary (*Im*) parts as follows:

(8) $$Re\left(\varepsilon \left(\omega \right)\right)=\varepsilon_{1}=1-\frac{\omega_{p}^{2}}{\omega^{2}+\omega_{\tau }^{2}}$$

and

(9) $$Im\left(\varepsilon \left(\omega \right)\right)=\varepsilon_{2}=\frac{\omega_{p}^{2}\omega_{\tau }}{\omega \left(\omega^{2}+\omega_{\tau }^{2}\right)}$$

Now, from Equation (6) the following expression for the extrapolated static conductivity, i.e., at ω =0, can be derived:

(10) $${Re\left(\sigma \right)=\sigma }_{1}=\varepsilon_{0}\omega \varepsilon_{2}=\varepsilon_{0}\frac{\omega_{p}^{2}}{\omega_{\tau }}$$

From the above Equations (9) and (10), it is clear that although $\varepsilon_{2}$ has a singularity at ω=0 (see Equation (9)), the electrical conductivity due to free carriers in the stationary state does not vanish. For completeness, let us add that the absorption coefficient, α, which can be determined from light transmission measurements, is directly proportional to the imaginary part of the dielectric function. More precisely, it can be expressed by the following formula:

(11) $$\alpha =\frac{\omega \varepsilon_{2}}{\varepsilon_{0}nc}$$

where *c* is the light velocity and *n* is the refractive index, which, in turn, is dependent on $\varepsilon_{1}$ and $\varepsilon_{2}$. Therefore, by fitting the PEDOT:PSS optical model to the optical transmittance, the Drude conductivity given in Equation (10) can be determined.

**Figure 4:**
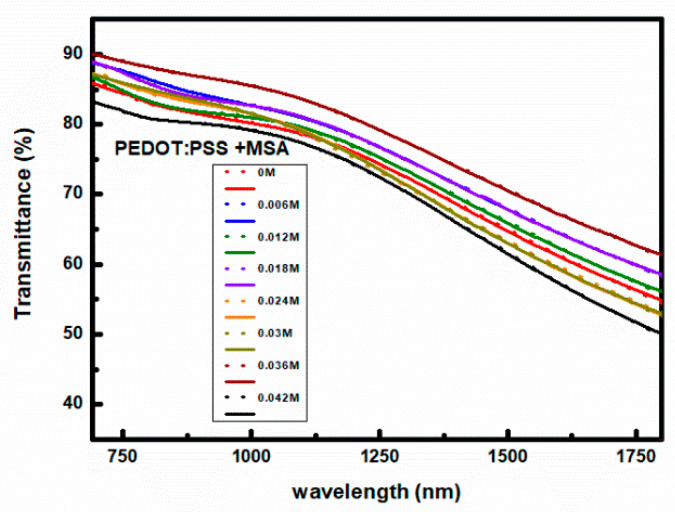
Optical transmittance of PEDOT:PSS films as a function of light wavelength for the indicated MSA doping level. Dots represent experimental points, and lines correspond to fits to the optical model described in the text.

**Figure 5:**
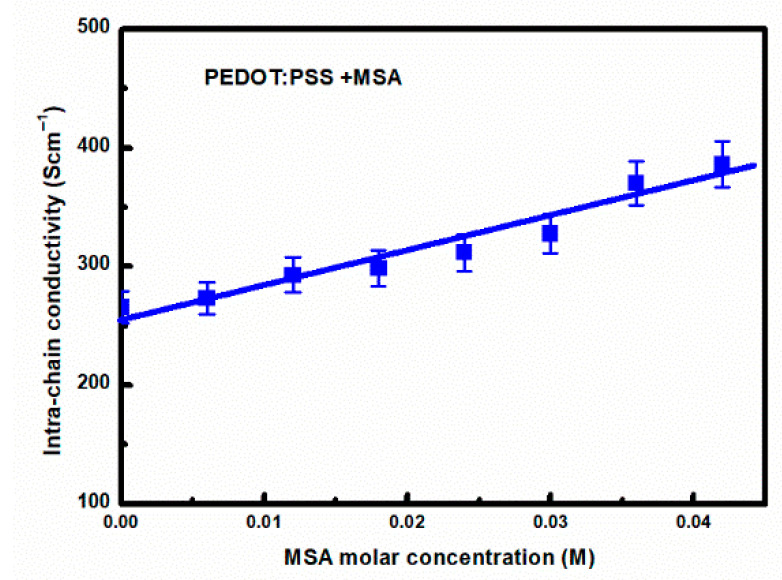
Intra-chain DC conductivity of PEDOT, determined from Equation (9), as a function of the MSA doping level.

Optical transmission studies of layers deposited on glass substrates were conducted in the spectral range of light from 350 to 2000 nm. Fitting the described optical model of PDOT:PSS layers to optical transmission measurements allows for the determination of its parameters. The results of such fitting for PEDOT:PSS thin films deposited from solutions with various MSA concentrations are shown in Figure 4. In the figure, the points represent experimental data, and the solid lines represent their fits. The quality of these five-parameter fits was controlled by the MSE, which was not greater than 0.1, see Table S3. Whereas, the PEDOT conductivity values calculated from Equation (9), i.e., extrapolated for ω = 0 according to Equation (9), are shown in Figure 5. In accordance with the above considerations, we interpret them as the intra-chain PEDOT conductivity. As can be seen in Figure 5, the intra-chain PEDOT DC conductivity shows a weak linear increase with increasing MSA doping.

### 3.4. Studies of Inter-Chain Charge Carrier Conduction in MSA Doped PEDOT:PSS Thin Films

**Figure 6:**
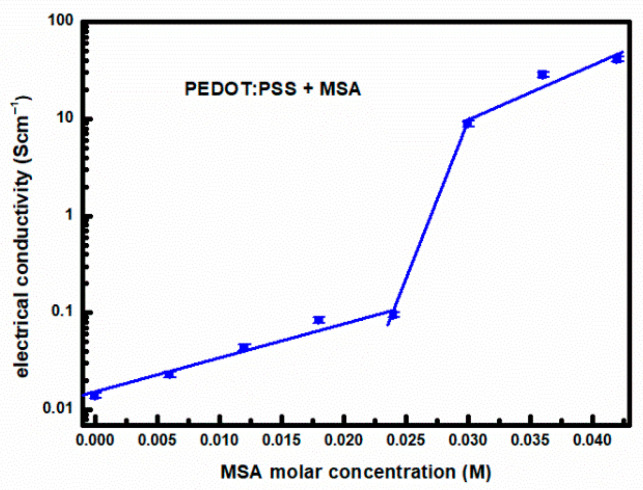
Electrical conductivity of PEDOT:PSS thin films as a function of MSA doping level, calculated from Equation (1), details in the text.

The electrical conductivity of PEDOT:PSS thin solid films as a function of MSA doping level, determined from surface resistivity measurements using the van der Pauw method on samples with an area of approximately 1 × 1 cm^2^, is presented in Figure 6.

**Figure 7:**
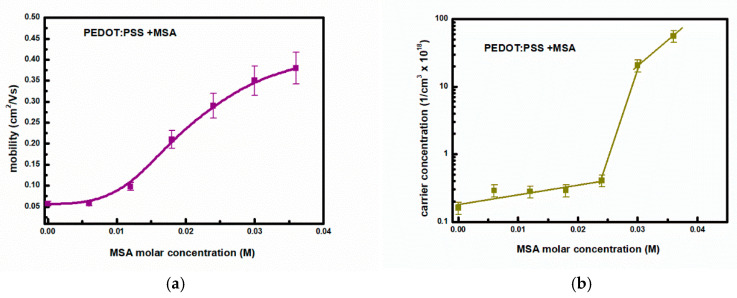
(**a**) Carrier mobility and (**b**) carrier concentration in PEDOT: PSS thin films as a function of MSA doping level.

As can be seen in Figure 6, the experimentally determined conductivity shows a sudden increase in the case of MSA doping of the aqueous dispersion of PEDOT:PSS (Clevios™ HTL Solar, Ossila M124) above the level of 0.024 M. Electrical conductivity measurements are supplemented with Hall effect measurements on the same samples, allowing determination of carrier concentration and mobility. These results are presented in Figure 7a,b, respectively. As seen in Figure 7b, the carrier concentration increases rapidly upon doping above 0.024 M with MSA. However, the mobility (Figure 7a) does not show such a jump in values. The accuracy of determining carrier mobility and density based on Hall effect measurements is significantly lower than that of determining film conductivity. This is due to the significantly lower signal-to-noise ratio (not exceeding 30).

The mechanisms by which the conductivity of PEDOT:PSS is increased by the addition of acid or organic solvent are still a matter of debate [30]. Nevertheless, some important results in this area require discussion. In the early stages of research on the electrical properties of PEDOT:PSS layers, Aleshin et al. [34] presented results on the conductivity of these layers as a function of the pH of the aqueous dispersion from which they were deposited, as well as a function of temperature. Conductivity studies at room temperature showed that it depends on the pH of the solution used to cast the layer. Temperature studies, in turn, showed that relation, $\sigma =\sigma_{0}exp\left(-{\left(T_{0}/T\right)}^{1/2}\right)$, is satisfied for all tested samples in the temperature range from 300 to 6 K, independent of pH. They also concluded that such temperature dependence is characteristic of granular metals, i.e., strongly disordered inhomogeneous systems, and identified the mechanism as the charging-energy limited tunneling model of Zuppiroli et al. [48].

**Figure 8:**
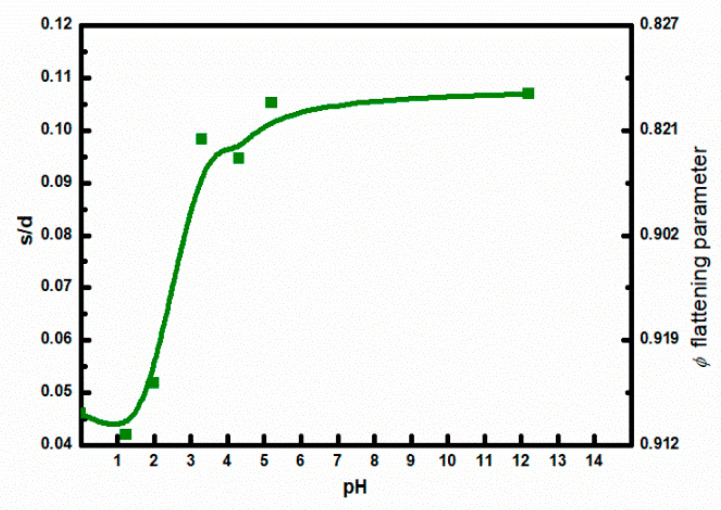
Ratio of the distance between PEDOT-rich grains to their diameter as a function of PEDOT:PSS solution pH determined on the base of results reported in [34].

This type of conduction, with temperature exponent ½ was also reported by Nardes et al. [49], for PEDOT:PSS films doped with organic solvents. It is important to note that the charging energy limited tunneling model relates $T_{0}$ to the ratio of the average distance between conducting PEDOT grains, *s*, to their average diameter, *d*, as follows [34,48]:

(12) $$T_{0}=\frac{8U}{k_{B}}{\left(\frac{s}{d}\right)}^{2}\frac{1}{\frac{1}{2}+\frac{s}{d}}$$

where U is the Coulomb repulsion energy of electrons on negatively charged site and $k_{B}$ is the Boltzman constant. These results provide additional confirmation that the pH of the PEDOT:PSS dispersion influences the microstructure of the PEDOT:PSS layers, especially that responsible for charge carrier transport between PEDOT-rich grains. Since Equation (12) was not used in [34] to determine the *s*/*d* ratio, its dependence on the solution pH is shown in Figure 8. Considering that the untreated PEDOT:PSS has a pH of 2, it can be concluded from Figure 8, that acid doping decreases the value of this ratio, while NaOH doping significantly increases it. Furthermore, from the increase in electrical conductivity with decreasing pH, we can conclude that the change in the *s/d* ratio is mainly caused by the decreasing distance between PEDOT-rich grains. For a typical PEDOT-rich grain size of 25 nm [49,50], the estimated minimum distance between them, at pH = 1.3, is about 1.05 nm. There is no doubt, however, that current conduction in untreated or only lightly doped PEDOT:PSS occurs via charge carrier hopping or tunneling between localized states. Therefore, in the model developed here, which treats PEDOT:PSS as a nanocomposite material, the PEDOT conductivity determined for free carriers based on the Drude model should be treated as the upper limit of achievable values after exceeding the percolation limit.

Section 3.1.2 in detail discusses the modeling of the electrical conductivity of PEDOT:PSS using a composite approach. It also argues that Bruggerman homogenization should be applied consistently to provide a coherent description of the optical and electrical properties of PEDOT:PSS layers. Therefore, it is natural to use it here to study the inter-chain conductivity in MSA-doped PEDOT:PSS thin films. The explicit form of BEMA for the electrical conductivity is as follows:

(13) $$\frac{\left(1-f\right)\left(\sigma_{PSS}-\sigma_{PEDOT:PSS}\right)}{L\sigma_{PSS}+\left(1-L\right)\sigma_{PEDOT:PSS}}+\frac{f\left(\sigma_{PEDOT}-\sigma_{PEDOT:PSS}\right)}{L\sigma_{PEDOT}+\left(1-L\right)\sigma_{PEDOT:PSS}}=0$$

where L denote the effective shape factor. This form of Equation (13) can accommodate values of L over the entire range from 0 to 1 and is valid for $\sigma_{PSS}=0$.

When applying the BEMA method to study the inter-chain conductivity in doped PEDOT:PSS layers, the following assumptions are made: For the onset of critical behavior, corresponding to a doping of approximately 0.03 M (see Figure 6), we use the following reasoning: two adjacent PEDOT:PSS gel particles in an approximate BEMA configuration are replaced by three spheres. The two representing the conducting PEDOT-rich cores are separated by a sphere representing the PSS inclusions. In this approach, layer deposition and/or doping deform the spheres, flattening them so that the spheroidal PEDOT inclusions come closer together, as shown in Figure S1 in the SM model. In this approach, percolation will occur when the elongated PEDOT spheroids come into contact with each other. According to the assumptions made, the ratio of the distance between PEDOT inclusions to their diameter, resulting from the change in radius is as follows:

(14) $$\frac{s}{d}=\frac{2\left(1-\varphi \right)R}{2\left(1+\varphi \right)R}$$

where φ is the flattening parameter describing the fractional change in the inclusion radius *R*. This gives the correct ratio *s/d* = 1 for *φ* = 0, and the spheres will be in contact for *φ* = 1. More details can be found in the Supplementary Materials.

Using Equation (14) for the results of Aleshin et al. [30], the value of the flattening parameter can be determined as a function of *s*/*d*, which is included on the right vertical axis in Figure 8.

**Figure 9:**
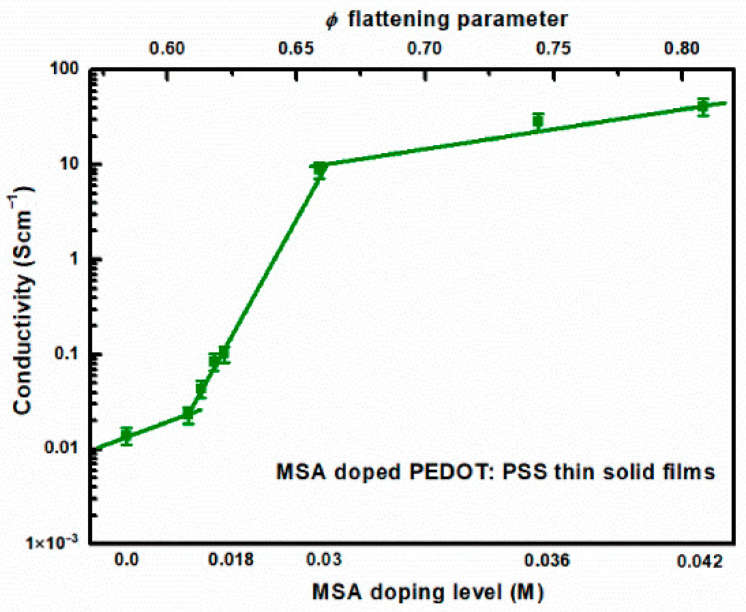
Conductivity as a function of flattening ratio and MSA doping level, calculated on the basis of Equation (11), details in the text.

The advantage of introducing the parameter *φ* is that it is related to the eccentricity of the ellipse formed by the cross-section through the axis of symmetry of the spheroid. This eccentricity, in turn, is used to determine the depolarization factor *L*. The mapping of the MSA doping level to the flattening parameter and its relationship to the depolarization factor *L* is presented in the SM. Taking all these factors into account allows the use of Equation (13) to determine the conductivity as a function of flattening parameter, what is shown in Figure 9. As seen in Figure 9, the inter-chain conductivity shows a steep increase in contrast to the intra-chain conductivity. This is not surprising, since, as expected, the inter-chain conductivity largely reflects the determined conductivity from sheet resistance measurement using the van der Pauw method, shown in Figure 6.

It should be emphasized that the interpretation presented here, which attributes acid doping to the flattening of PEDOT:PSS gel nanoparticles, is well supported by the results of dynamic light scattering (DLS) studies on aqueous PEDOT:PSS dispersion, which show that the size of PEDOT:PSS gel particles depends on the solution pH, and their diameter changes from 12 to 19 nm when the solution pH changes from 1.7 to 9 [46]. On the other hand, the studies on the morphology of PEDOT:PSS layers conducted by Nardes et al. [49,50] clearly prove their flattened shape and lamellar, alternating arrangement of PEDOT-rich and PSS-rich layers.

## 4. Conclusions

We investigated the optical and electrical properties of PEDOT:PSS thin films deposited on glass substrates via spin-coating of aqueous PEDOT:PSS doped with MSA. The goal was to determine the effect of MSA doping of PEDOT:PSS thin films on the intra-chain and inter-chain conductivity. For this purpose, we revised and significantly extended the existing model [40], which now treats PEDOT:PSS as a nanocomposite material. In particular, we justified the linear mixing of the dielectric functions of the nanocomposite components, deriving it as a limiting case of the Bruggerman effective medium approximation due to the resolution limitations of optical measurements in the case of the complex hierarchical microstructure of PEDOT:PSS thin films. Furthermore, to maintain consistency in the description of optical and electrical properties, we assumed that the electrical conductivity should also be determined based on Bruggerman homogenization rather than GEMT. Acid doping of PEDOT:PSS films was modeled using a depolarization factor accounting for the inclusion shape, which also allowed for the conductivity percolation phenomenon to be taken into account using a flattening parameter. This model was then used to investigate the effect of MSA doping on the intra-chain and inter-chain conductivity of PEDOT:PSS films. Our approach quantitatively attributes the high free carrier conductivity from PEDOT-rich cores to intra-chain carrier transport using parameters derived from the optical Drude-Lorentz model. The lower inter-chain conductivity between PEDOT-rich grains is modeled as being associated with the poor conductivity of PSS. Our studies show that MSA doping significantly affects both types of conductivity. The intra-chain conductivity of PEDOT increases from 260 to almost 400 Scm^−1^. Meanwhile, the inter-chain conductivity increases by almost three orders of magnitude, confirming that the percolation threshold has been exceeded. The observed changes in electrical conductivity due to acid doping are attributed to the flattening of the PEDOT:PSS gel nanoparticles. In the model developed here, this flattening is accounted for by the inclusion of the shape factor.

## Data Availability

The original contributions presented in this study are included in the article and Supplementary Materials. Further inquiries can be directed to the corresponding author.

## Supplementary Materials

The following supporting information can be downloaded at: https://www.mdpi.com/article/10.3390/ma18194569/s1, Figure S1: Modeling of the influence of MSA doping of PEDPT:PSS thin films on shape of the PEDOT:PSS gel particles; Table S1: A summary of the film preparation parameters and MSA concentrations; Table S2: thickness of the tested films; Table S3: PEDOT:PSS optical model fitted parameter values.
