# Supplementary material for: Studies of Intra-Chain and Inter-Chain Charge Carrier Conduction in Acid Doped Poly(3,4-ethylenedioxythiophene) Polystyrene Sulfonate Thin Films
Source: Materials (Basel). 2025 Oct 1;18(19):4569. doi: 10.3390/ma18194569 (PMC12525955; doi:10.3390/ma18194569)
Supplement: Supplementary file 1 [file materials-18-04569-s001.zip › materials-3866178-supplementary.pdf]

Supplementary Materials

Studies of Intra-Chain and Inter-Chain Charge Carrier Conduction in Acid Doped Poly(3,4-ethylenedioxythiophene) Polystyrene Sulfonate Thin Films

Ayman A. A. Ismail [1,2], Henryk Bednarski[1,*] and Andrzej Marcinkowski [1]

[1]Centre of Polymer and Carbon Materials, Polish Academy of Sciences, M. Curie-Skłodowskiej 34, 41-819 Zabrze, Poland

[2]Joint Doctoral School, Silesian University of Technology, Akademicka 2a, 44-100 Gliwice, Poland; aabdallah@polsl.pl

*Correspondence: hbednarski@cmpw-pan.pl

Contents:

1. Table 1S – A summary of the film preparation parameters and MSA concentrations
2. Table 2S thickness of the films
3. Table S3 – Parameter values used for the PEDOT:PSS optical model
4. Table S 4 AFM Rq Values (nm) for Various MSA Concentrations in PEDOT:PSS
5. Figure S1 – Mapping of the doping level to the flattening parameter – model of influence of MSA doping on the shape of PEDOT-reach grains

1. The table below summarizes the film preparation parameters, including the MSA concentration in the solution used to prepare the film.

1.Table S1. A summary of the film preparation parameters and MSA concentrations

Sample ID	PEDOT:PSS Volume (µL) ±1	MSA Volume (µL) ±0.1	MSA Concentration in PEDOT:PSS (M)	Spin Coating Protocol	Thermal Treatment	Electrode Type
S0	165	0.0	0	500 rpm, 3 s → 3000 rpm, 3 s	120 °C, 5 min → 65 °C, 5 min	Silver Paste
S1	165	1	0.006	500 rpm, 3 s → 3000 rpm, 3 s	120 °C, 5 min → 65 °C, 5 min	Silver Paste
S2	165	2	0.012	500 rpm, 3 s → 3000 rpm, 3 s	120 °C, 5 min → 65 °C, 5 min	Silver Paste
S3	165	3	0.018	500 rpm, 30s → 3000 rpm, 3 s	120 °C, 5 min → 65 °C, 5 min	Silver Paste

**S4**	**165**	**4**	**0.024**	**500 rpm, 3 s → 3000 rpm, 3 s**	**120 °C, 5 min → 65 °C, 5 min**	**Silver Paste**
**S5**	**165**	**5**	**0.030**	**500 rpm, 3 s → 3000 rpm, 3 s**	**120 °C, 5 min → 65 °C, 5 min**	**Silver Paste**
**S6**	**165**	**6**	**0.036**	**500 rpm, 30s → 3000 rpm, 3 s**	**120 °C, 5 min → 65 °C, 5 min**	**Silver Paste**
**S7**	**165**	**7**	**0.042**	**500 rpm, 30s → 3000 rpm, 3 s**	**120 °C, 5 min → 65 °C, 5 min**	**Silver Paste**

2. Table 2S contains the thickness of the tested films determined by the AFM method.

2.Table 2S thickness of the tested films

**Sample ID**	**MSA Concentration in PEDOT: PSS (M)**	**Thickness ±15%**
S0	0	150
S1	0.006	160
S2	0.012	140
S3	0.018	160
S4	0.024	140
S5	0.030	150
S6	0.036	120
S7	0.042	160

3. Table S3 contains parameter values used for the PEDOT:PSS optical model.

3.Tabl S3 PEDOT:PSS optical model fitted parameter values

S0

Variable parameters of optical model (without thicknesses)

Name	Value	Error	
p2msa7: Inclusion:w-p-free-carriers-(1/cm)	15799.24	162.756	1%
p2msa7: Inclusion:w-tau-free-carriers-(1/cm)	2493.65	145.806	6%
p2msa7: Inclusion:(3)Omega-O-(1/cm)	12302.22	232.342	2%
p2msa7: Inclusion:(3)Omega-p-(1/cm)	3645.35	493.547	14%
p2msa7: Inclusion:(3)Omega-tau-(1/cm)	6688.13	665.053	10%

S1

Variable parameters of optical model (without thicknesses)

Name	Value	Error		Comment
p2msa7: Inclusion:w-p-free-carriers-(1/cm)	16276.80	180.747	1%	
p2msa7: Inclusion:w-tau-free-carriers-(1/cm)	2574.46	146.773	6%	
p2msa7: Inclusion:(3)Omega-O-(1/cm)	11160.42	253.362	2%	
p2msa7: Inclusion:(3)Omega-p-(1/cm)	2162.49	584.354	27%	error > 20%
p2msa7: Inclusion:(3)Omega-tau-(1/cm)	3831.51	1061.416	28%	error > 20%

S2

Variable parameters of optical model (without thicknesses)

Name	Value	Error	
p2msa7: Inclusion:w-p-free-carriers-(1/cm)	16425.51	178.365	1%
p2msa7: Inclusion:w-tau-free-carriers-(1/cm)	2445.69	149.101	6%
p2msa7: Inclusion:(3)Omega-O-(1/cm)	11978.10	151.647	1%
p2msa7: Inclusion:(3)Omega-p-(1/cm)	3502.43	440.850	13%
p2msa7: Inclusion:(3)Omega-tau-(1/cm)	5135.32	557.021	11%

S3

Variable parameters of optical model (without thicknesses)

Name	Value	Error	
p2msa7: Inclusion:w-p-free-carriers-(1/cm)	16829.15	185.297	1%
p2msa7: Inclusion:w-tau-free-carriers-(1/cm)	2518.39	147.087	6%
p2msa7: Inclusion:(3)Omega-O-(1/cm)	11455.59	153.693	1%
p2msa7: Inclusion:(3)Omega-p-(1/cm)	2606.18	456.728	18%
p2msa7: Inclusion:(3)Omega-tau-(1/cm)	3718.65	637.880	17%

S4

Variable parameters of optical model (without thicknesses)

Name	Value	Error	
p2msa7: Inclusion:w-p-free-carriers-(1/cm)	17864.97	199.238	1%
p2msa7: Inclusion:w-tau-free-carriers-(1/cm)	2585.90	181.148	7%
p2msa7: Inclusion:(3)Omega-O-(1/cm)	12332.21	178.285	1%
p2msa7: Inclusion:(3)Omega-p-(1/cm)	2591.29	505.439	20%
p2msa7: Inclusion:(3)Omega-tau-(1/cm)	3845.65	690.677	18%

S5

Variable parameters of optical model (without thicknesses)

Name	Value	Error		Comment
p2msa7: Inclusion:w-p-free-carriers-(1/cm)	17034.04	162.824	1%	
p2msa7: Inclusion:w-tau-free-carriers-(1/cm)	2469.38	146.177	6%	
p2msa7: Inclusion:(3)Omega-O-(1/cm)	12519.80	325.312	3%	
p2msa7: Inclusion:(3)Omega-p-(1/cm)	2617.62	593.182	23%	error > 20%
p2msa7: Inclusion:(3)Omega-tau-(1/cm)	5571.67	995.411	18%	

S6

Variable parameters of optical model (without thicknesses)

Name	Value	Error		Comment
p2msa7: Inclusion:w-p-free-carriers-(1/cm)	17636.45	277.609	2%	
p2msa7: Inclusion:w-tau-free-carriers-(1/cm)	2225.27	197.580	9%	
p2msa7: Inclusion:(3)Omega-O-(1/cm)	11886.08	241.799	2%	
p2msa7: Inclusion:(3)Omega-p-(1/cm)	2511.53	596.292	24%	error > 20%
p2msa7: Inclusion:(3)Omega-tau-(1/cm)	4506.33	971.089	22%	error > 20%

S7

Variable parameters of optical model (without thicknesses)

Name	Value	Error		Comment
p2msa7: Inclusion:w-p-free-carriers-(1/cm)	17086.49	161.602	1%	
p2msa7: Inclusion:w-tau-free-carriers-(1/cm)	2005.64	81.613	4%	
p2msa7: Inclusion:(3)Omega-O-(1/cm)	13006.76	120.400	1%	
p2msa7: Inclusion:(3)Omega-p-(1/cm)	5201.81	443.228	9%	
p2msa7: Inclusion:(3)Omega-tau-(1/cm)	8208.87	447.717	5%	

It should be noted that the errors in determining the free carrier parameters are relatively low for all samples.

4. Table S4 summarizes the values of the root mean square roughness parameters Rq determined by the AFM method.

4. Tabl S 4 AFM Rq nm FOR MSA concentrations in the PEDOT: PSS

**Rq nm**	**MSA concentrations in the PEDOT: PSS**
4.93	0
2.68	0.006
2.49	0.012
2.44	0.018
2.21	0.024
2.02	0.030
1.35	0.036
1.76	0.042

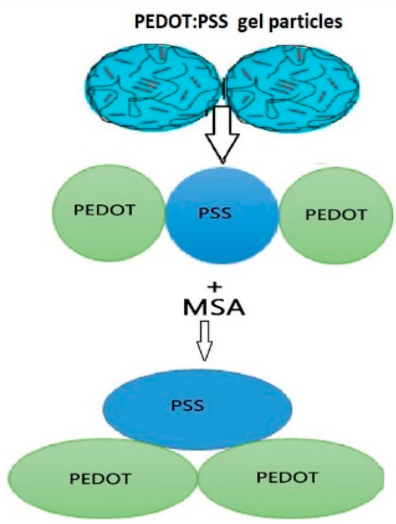

Figure S1. Modeling of the influence of MSA doping of PEDPT:PSS thin films on shape of the PEDOT:PSS gel particles.

5. Mapping of the doping level to the flattening parameter – model of influence of MSA doping on the shape of PEDOT-reach grains.

Details regarding the relationship between doping level and effective depolarization coefficient are presented here. An illustration of the proposed nanoscopic doping model for PEDOT:PSS, discussed in the main text, is shown in Figure 1S. Because PEDOT:PSS layers exhibit uniaxial anisotropy, with the optical axis directed perpendicular to the layer surface, a 2D graphic is sufficient. In the adopted model, doping the aqueous dispersion with acid causes plasticization of the PEDOT:PSS gel particles, which can undergo further flattening during spin deposition. As shown in the upper part of Figure S1, the middle figure shows an EMA model of this situation using three unit cells with probability f that it is PEDOT and (1-f) that it is PSS. To quantitatively describe this deformation, consider an oblate sphere preserving uniaxial anisotropy (and volume), as shown in the lower part of Figure S1. Let us therefore suppose that in the direction of the optical axis we contract the radius of the sphere, *R*, by a fraction of

its length $R'=R/(1+\varphi)^2$. This causes the radius of the sphere in the equatorial plane to increase to $R''=R(1+\varphi)$. We now see that these spheroids will touch under the assumed conditions, then s, i.e., distance between two PEDOT-reach grains, described by Equation (14) will be equal to 0, and therefore for φ equal to 1. What is important, the eccentricity, e, of the corresponding ellipse is described by the following equation

$$e(\varphi) = \sqrt{1 - \left(\frac{1}{(1+\varphi)^3}\right)^2} \qquad \text{S1}$$

In turn, corresponding depolarization factor, for an oblate spheroid along the rotational axis is expressed as follows [1]:

$$L = \frac{1}{e^2}\left[1 - \frac{(1-e^2)^{\frac{1}{2}}}{e}\arcsin(e)\right] \qquad \text{S2}$$

Note that relation between parallel and serial resistance equivalent circuit for the Equation (13) is effected using the following transformation:

$$e_{\parallel} + e_{\perp} = 1$$

Which in terms of the inclusion shape corresponds to the switching between oblate and prolate spheroids with rotational axis oriented parallel to the optical axis of the film.

References

[1] Yunos NM, Zubir MNM, Rahman NA, Ahmad N, Mohamed NS. The depolarization factors for ellipsoids and some of their properties. *Malays J Fundam Appl Sci.* 2019;15(6):784-789.
